# Health literacy in childhood and youth: a systematic review of definitions and models

**DOI:** 10.1186/s12889-017-4267-y

**Published:** 2017-04-26

**Authors:** Janine Bröder, Orkan Okan, Ullich Bauer, Dirk Bruland, Sandra Schlupp, Torsten M. Bollweg, Luis Saboga-Nunes, Emma Bond, Kristine Sørensen, Eva-Maria Bitzer, Susanne Jordan, Olga Domanska, Christiane Firnges, Graça S. Carvalho, Uwe H. Bittlingmayer, Diane Levin-Zamir, Jürgen Pelikan, Diana Sahrai, Albert Lenz, Patricia Wahl, Malcolm Thomas, Fabian Kessl, Paulo Pinheiro

**Affiliations:** 1 Centre for Prevention and Intervention in Childhood and Adolescence CPI, Bielefeld University, Bielefeld, Germany; 2 National School of Public Health, Universidade NOVA de Lisboa, Lisbon, Portugal; 3 University of Suffolk, Ipswich, UK; 4 Global Health Literacy Academy, Urmond, The Netherlands; 5 University of Education, Freiburg i.Br, Germany; 6 Robert Koch Institute, Berlin, Germany; 7 CIEC, Institute of Education, University of Minho, Braga, Portugal; 8 School of Public Health, University of Haifa, Haifa, Israel; 9 Austrian Public Health Institute, Gesundheit Österreich GmbH, Wien, Austria; 10 School of Education, Basel, Switzerland; 11 Katholische Hochschule Nordrhein-Westfalen, Paderborn, Germany; 12 School of Education and Lifelong Learning, Aberystwyth University, Aberystwyth, UK; 13 University Duisburg-Essen, Essen, Germany

**Keywords:** Health Literacy, Health Competencies, Children, Young People, Adolescents, Pupils, Definitions, Conceptual Models, Dimensions, Literature Review

## Abstract

**Background:**

Children and young people constitute a core target group for health literacy research and practice: during childhood and youth, fundamental cognitive, physical and emotional development processes take place and health-related behaviours and skills develop. However, there is limited knowledge and academic consensus regarding the abilities and knowledge a child or young person should possess for making sound health decisions. The research presented in this review addresses this gap by providing an overview and synthesis of current understandings of health literacy in childhood and youth. Furthermore, the authors aim to understand to what extent available models capture the unique needs and characteristics of children and young people.

**Method:**

Six databases were systematically searched with relevant search terms in English and German. Of the *n* = 1492 publications identified, *N* = 1021 entered the abstract screening and *N* = 340 full-texts were screened for eligibility. A total of 30 articles, which defined or conceptualized generic health literacy for a target population of 18 years or younger, were selected for a four-step inductive content analysis.

**Results:**

The systematic review of the literature identified 12 definitions and 21 models that have been specifically developed for children and young people. In the literature, health literacy in children and young people is described as comprising variable sets of key dimensions, each appearing as a cluster of related abilities, skills, commitments, and knowledge that enable a person to approach health information competently and effectively and to derive at health-promoting decisions and actions.

**Discussion:**

Identified definitions and models are very heterogeneous, depicting health literacy as multidimensional, complex construct. Moreover, health literacy is conceptualized as an action competence, with a strong focus on personal attributes, while also recognising its interrelatedness with social and contextual determinants. Life phase specificities are mainly considered from a cognitive and developmental perspective, leaving children’s and young people’s specific needs, vulnerabilities, and social structures poorly incorporated within most models. While a critical number of definitions and models were identified for youth or secondary school students, similar findings are lacking for children under the age of ten or within a primary school context.

## Background

From a public health perspective, children and young people constitute a core target group for health literacy research and intervention as during childhood and youth, fundamental cognitive, physical and emotional development processes take place [1] and health-related behaviours and skills develop. As a result, these stages of life are regarded as crucial for healthy development, as well as for personal health and well-being throughout adulthood [2, 3]. Moreover, health literacy is understood as a variable construct that is acquired in a life-long learning process, starting in early childhood [4]. Hence, targeting children and young people with health literacy interventions can help promoting healthy behaviors and ameliorate future health risks.

Whilst we acknowledge the recent increase in publications which focus on children and young people, the attention contributed to children’s and young people’s health literacy is still small compared to the momentum health literacy is currently experiencing in research, practice and policy-making. Within health care settings, research has mainly focused on the impact of parental or maternal health literacy on children’s health. Accordingly, most research primarily addresses questions of how children are affected when their parents lack the knowledge and skills required for making sound health decisions concerning their children’s health [5]. Within health promotion, some attention has been drawn to addressing children’s and young people’s health literacy in school health education and health promotion [6–9].

Moreover, there is limited knowledge and academic consensus regarding the abilities and knowledge a child or young person should possess for making sound health decisions. For the general population, the European Health Literacy Consortium integrated both drivers and differing dimensions to suggest: health literacy is “linked to literacy and entails people’s knowledge, motivation and competences to access, understand, appraise, and apply health information in order to make judgments and take decisions in everyday life concerning healthcare, disease prevention and health promotion to maintain or improve quality of life during the life course.” [10]. Moreover, an individual’s health literacy depends upon their personal situation including their health status, risks or problems, their affiliation with social group(s) (e.g. health practitioners, patients, and different age-groups) and other socio-economic determinants [11]. A more specific overview for child and youth health literacy is lacking. Hence, it is unclear, to what extent conceptual and theoretical efforts for shaping and describing health literacy in children and young people currently do consider the unique characteristics of the target group and recognise related challenges. Rothman et al. [12] proposed four categories of unique needs and characteristics to contrast the target group from the general adult population, namely (1) developmental changes, (2) dependency on resources and skills, (3) epidemiological differences, and (4) vulnerability to social-demographic determinants of health.

To address these described research gaps, this article aims:

- *to scope current understandings of health literacy in childhood and youth and*
- *to understand to what extent available models capture the unique needs and characteristics of children and young people.*

For this purpose, a systematic review and inductive content analysis of health literacy definitions and models for persons aged 18 or younger was conducted in English and German academic literature. To the authors’ knowledge, this work is the first to scope the conceptual understanding of health literacy in children and young people in a systematic manner. The research is conducted in the context of the German Health Literacy in Childhood and Adolescence (HLCA) Consortium [13] and seeks to provide a first step towards future effective health literacy interventions to promote children’s and young people’s health.

## Method

A systematic review of available generic health literacy definitions and models for children and young people aged 18 or younger was conducted in accordance with the Preferred Reporting Items for Systematic Reviews and Meta-Analyses (PRISMA) guidelines for ensuring high quality and transparent reporting of reviews [14]. Within this research, health literacy is regarded as a multidimensional construct for which the available latest research is being synthesized and evaluated. Hence, it consists of multiple underlying dimensions that entail the generalizable characteristics of health literacy. Health literacy dimensions were extracted from available conceptual models. These were clustered according to their stated purpose as conceptualisation – the process by which imprecise constructs and their constituent dimensions are defined in concrete terms – or operationalisations, which provide the base for measuring the construct or testing it with defined variables [15].

### Search strategy and screening process

Between May - Nov. 2015, six bibliographic databases were searched, including PubMed, the Educational Resources Information Centre (ERIC), the Cumulative Index to Nursing and Allied Health Literature (CINAHL), PsycINFO, Web of Science, in English and the FIS Bildung Literaturdatenbank in German. Search terms in English and German were defined for three distinctive search clusters - main topic, subtopic, and target population (see Table 1) - and were selected upon a narrative search. Search terms were combined through Boolean operators (AND/OR) and truncations and wildcard characters were used to increase the sensitivity of the searches. The searches were not limited to any publication time frame, research design or peer-review criteria (dissertations and essays were included). Theory-building or conceptual, explorative publication are often part of an inductive research process, providing the theoretical base for hypothesis-testing research. Therefore, not all quality standards as outlined in the PRISMA guidelines applied to our research question.

**Table 1:** Search terms for systematic literature search

Topic		**And**	Sub-topic
English:	Health literacy, “health literacy”, health competence		Skill*, competen*, concept*, theor*, model*, framework*, Defin*
			Fähigkeit*, Kompetenz*, Konzept*, Theorie*, Model*, Rahmen*, Definition*
			**OR**
German:	Gesundheitskompetenz, −mündigkeit, −bildung		Target population
			Child*, adolescen*, youth, teen* pupil*, student*
			Kind*, Jugend*, Teen*, Schüler*

Notes: Examples for a combination of search terms, in English: (health literacy (health AND literacy) OR health competence) OR (skill* OR competenc*) AND (child*OR adolescen* OR youth* OR teen*) AND (concept* OR theor* OR model* OR framework OR defin*)

In German: (Gesundheitskompetenz (Gesundheit AND Kompetenz) OR Gesundheitsmündigkeit OR Gesundheitsbildung OR Fähigkeit) AND (Kind* OR Jugend*OR Heranwachsend* OR Teen*) AND (Konzept* OR Theor* OR Model* OR Rahmen* OR Defin*)

**Fig. 1:**
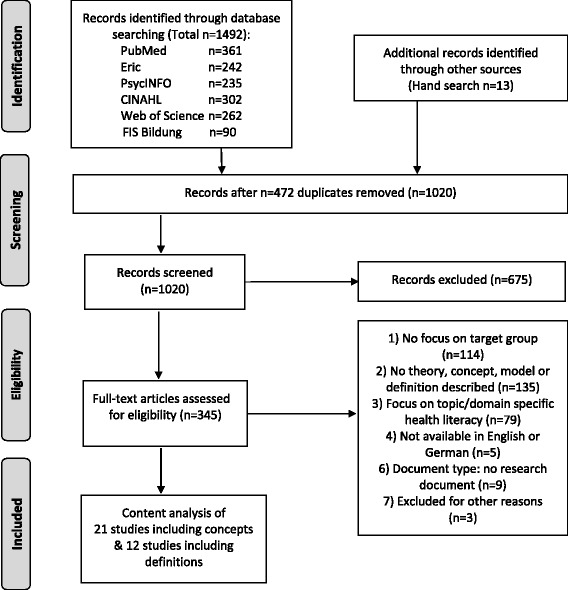
PRISMA chart of systematic search process

The search identified *n* = 1492 publications (see PRISMA Chart in Fig. 1). After removing duplicates (*n* = 471), 1020 abstracts were screened by JB and OO. Database searches were complemented by hand searches, e.g. in Google Scholar, and a cross-check of the reference lists of studies included for analysis, retrieving 13 additional articles that entered the selection process.

Articles were eligible if they: a) were fully available in English or German; b) focused on generic health literacy – while excluding any domain or topic-specific health literacy models, to ensure a focus on the core dimensions of health literacy [16]; c) offered relevant content for defining and conceptualizing health literacy in children and young people and d) addressed a target population that were 18 years or younger. Articles incorporating a life course perspective on health literacy were included as well. The life course concept refers to the sequence of age categories that people normally pass through as they develop and progress from birth to death. Thus, even without specifically stating the target group, the life course concept specifically encompasses children and young people as well. Therefore, the articles were included as they added to the comprehensiveness and the entirety of the analysis.

Whether articles were included for full-text analysis was determined by JB and SS based on the articles’ assessed fit with the eligibility criteria. Publications, for which the researchers reached a differential decision, were discussed within the core research team and if necessary assessed again until consensus was reached.

### Data extraction and analysis

The 30 articles were selected for the full-text analysis for the following reasons: four contained only a definition [1, 17–19], thirteen only a model [5, 20–31] and eight described a definition and a model [4, 8, 10, 32–36]. For three models two original references [37–39] were included as both provided additional insights to the model. All these articles were qualitatively assessed and synthesized applying a four-step inductive content analysis. Firstly, eligible publications were scanned for definitions and conceptual models that were either developed for the target group or adapted to it, or included relevant perspectives on health literacy for children and young people. Secondly, these definitions and models were coded and extracted by the research team following an inductive approach. Overlapping definitions and models from the same research group, were only included once. For non-related publications that described the same health literacy definitions or models, only the original reference was included and marked accordingly in Table 3. Thirdly, relevant background variables were defined and extracted into a matrix. These background variables included the age of the target group, the reasons for focussing on the target group, whether the target groups’ perspectives were considered (a) in the development of the definition or model, or (b) in the applicability and relevance of these, and the setting(s) for which they were developed. The articles’ research design and methodological quality was not assessed as many studies were theoretical explorations for which the assessment criteria of PRISMA did not apply. Finally, the definitions and identified dimensions were discussed with a whole research team in autumn 2015 and the feedback was integrated into the final analysis.

## Results

The systematic review of the literature identified 12 definitions (Table 2) and 21 models (Table 4) of health literacy for children and young people.

**Table 2:** Definitions of children’s and young people’s health literacy

Children & Primary School Students		
A	Fok & Wong [17]	The meaning of health literacy to children is defined as “to understand and act upon physical and psycho-social activities with appropriate standards, being able to interact with people and cope with necessary changes and; demands reasonable autonomy so as to achieve complete physical, mental and social well-being.
B	Brown et al. [32]	“For this study, health literacy was defined simply as the ability to understand health information and to understand that actions taken in youth affect health later in life, combined with the ability to access valid health information.”
Young people & Secondary School Students		
C	Massey et al. [33]	“We take an expanded perspective of health literacy and define it as a set of skills used to organize and apply health knowledge, attitudes and practices relevant when managing one’s health environment.”
D	Paakkari & Paakkari [8]	“Health Literacy is defined in the following terms: Health literacy comprises a broad range of knowledge and competencies that people seek to encompass, evaluate, construct and use. Through health literacy competencies people become able to understand themselves, others and the world in a way that will enable them to make sound health decisions, and to work on and change the factors that constitute their own and others’ health chances.”
E	Wu et al. [18]	“Health literate individuals are able to understand and apply health information in ways that allow them to take more control over their health through, for example, appraising the credibility, accuracy and relevance of information and acting on that information to change their health behaviours or living conditions.”
F	Gordon et al. [19]	“Health Literacy is the degree to which individuals have the capacity to obtain, access, process, and understand basic health information and services needed to take appropriate health decisions and involves an ongoing process of building individual and community capacity to understand the components of health.”
Different age groups or considering a life course perspective		
G	Borzekowski [1]	“Health literacy is not just the ability to read, rather, it is a set of skills that involve recognizing, processing, integrating, and acting on information from a variety of platforms. Those between the ages of 3 and 18 can seek, comprehend, evaluate, and use health information, especially if materials are presented in ways that are age appropriate, culturally relevant, and socially supported. The development of health literacy among children and young people can empower this vulnerable and “marginalized” group to be more engaged, more productive, and healthier.”
H	Soellner et al. [36]	[Translated] The working definition defines health competences (Gesundheitskompetenz) as an accumulation of skills and capabilities that someone has at one’s command to be able to act in daily life and in dealing with the health system, in such a ways that positively affect one’s health and well-being.
I	Mancuso [34]	“A process that evolves over one’s lifetime and encompasses the attributes of capacity, comprehension, and communication. The attributes of health literacy are integrated within and preceded by the skills, strategies, and abilities embedded within the competencies needed to attain health literacy. The outcomes of health literacy are dependent upon whether one has achieved adequate or inadequate health literacy and have the potential to influence individuals and society.”
J	Nutbeam [35]	“The personal, cognitive and social skills which determine the ability of individuals to gain access to, understand, and use information to promote and maintain good health”
K	Sørensen et al. [10]	“Health literacy is linked to literacy and entails people’s knowledge, motivation and competences to access, understand, appraise, and apply health information in order to make judgments and take decisions in everyday life concerning healthcare, disease prevention and health promotion to maintain or improve quality of life during the life course.”
L	Zarcadoolas, Pleasant & Greer [20]	“Health literacy evolves over one’s life course, starting at an early age, and, like most complex human competencies, is impacted by health status as well as demographic, socio-political, psychosocial and cultural factors.” “We define health literacy as the wide range of skills, and competencies that people develop to seek out, comprehend, evaluate and use health information and concepts to make informed choices, reduce health risks and increase quality of life.”

### Definitions of health literacy in childhood and youth

Of the 12 definitions (Table 2), two specifically targeted children younger than 12 [17, 32], another one included children from 3 to 18 years [1]. Four definitions focused on young people at different ages between 13 and 18 years [8, 18, 19, 33], while five articles considered health literacy over the life course without specifying a target group [10, 20, 34–36]. Four definitions were developed from a school health education perspective [8, 18, 19, 32]. While Massey [33] specifically targeted health literacy in the health care setting, Mancuso [34] and Sørensen et al. [10] stressed the relevance of health literacy in multiple health-related settings including health care, disease prevention, health promotion, and public health. The definitions by Fok and Wong [17] and Massey et al. [33] were the only ones where the target group participated in the development of empirical and explorative dimensions. Gordon et al. [19] developed the definition as a result of a stakeholder consultation with school health community partners and Sørensen et al. [10] evolved from the results of their systematic literature review.

The inductive narrative synthesis [40] of the definitions revealed seven content categories: (1) components, namely skills, abilities, competences, etc., (2) actions or agency, (3) subjects, (4) sources of information, (5) purpose, (6) conditions, (7) time perspective (see Table 3).

**Table 3:** Results from the inductive content analysis of definitions

Components: • collection/set of skills (*n* = 6) • competencies (*n* = 5) • knowledge (*n* = 3) • capacity (*n* = 2)	• motivation (*n* = 1) • strategies (*n* = 1) • comprehension (*n* = 1) • communication (*n* = 1)
Action/Agency: • to understand (*n* = 5) • to access (*n* = 4) • to use (*n* = 4) • to apply (*n* = 3) • to comprehend (*n* = 3) • to evaluate (*n* = 3) • to act (upon) (*n* = 3) • to seek (out) (*n* = 2)	• to appraising (*n* = 2) the credibility, accuracy and relevance of • to process (*n* = 2), • to obtain (*n* = 1) • to encompass (*n* = 1) • to integrate (*n* = 1) • to construct (*n* = 1) • to interact with people (*n* = 1) • to cope with necessary changes (*n* = 1) • to organize (*n* = 1)
Subjects: • (basic) health information (*n* = 7) • health-related concepts (*n* = 1) • health services (*n* = 1)	• health knowledge, attitudes and practice (*n* = 1) • physical and psycho-social activities with appropriate standards (*n* = 1)
Sources: from a variety of platforms (*n* = 1)	
Purposes: • to take/make appropriate/sound health decisions (*n* = 3) (concerning healthcare, disease prevention and health promotion) • to make informed choice (*n* = 1) • to manage one’s health environment (*n* = 2) • to maintain or improve quality of life (*n* = 2) • to improve or achieve complete well-being (*n* = 2) to promote and maintain good health (*n* = 1) • to make judgments (*n* = 1)	• to take more control over their health (*n* = 1) • to understand themselves, others and the world (*n* = 1) • to reduce health risks (*n* = 1) • to change their health behaviours or living conditions (*n* = 1) • to empower this group to be more engaged, more productive, and healthier (*n* = 1) • to build individual and community capacity to understand the components of health (*n* = 1) • the potential to influence individuals and society (*n* = 1) • to understand that actions taken in youth affect health later in life (*n* = 1)
Age- and development-specific conditions: • if materials are presented in ways that are age appropriate, culturally relevant, and socially supported (*n* = 1) • demands reasonable autonomy (*n* = 1)	
Time: • ongoing process (*n* = 2) • evolves over one’s lifetime (*n* = 2)	• during the life course (*n* = 1) • starting at an early age(*n* = 1)

Although diversely defined, health literacy was commonly portrayed as an individual-based construct, with a multidimensional nature [20]. As such, it exceeds basic reading, writing or numerical abilities [1]. In addition, health literacy entails a combination of different health-related skills, competences, and knowledge, as well as a motivational component that an individual possesses [10].

All definitions share a dominant demand or action-related focus mostly directed towards the access, process and application of health information [10]. These actions entail immediate cognitive or behavioural tasks that health literate persons should be able to perform when encountering situations that demand health-related decision-making in daily life or within the health care context. As such, children and young people are viewed as actors that actively and deliberately participate in seeking, processing, and evaluating health information (as well as health services, knowledge, attitudes and practices). The acquired information can be adequately used in health-informed decision-making, which form a direct output dimension for observing or measuring children’s or young people’s health literacy [8]. Fok and Wong [17] focus not only on health information-related tasks but on actions related to physical and psycho-social activities children engage in. They point out that children are health literate once they understand how to achieve health and well-being by obtaining certain attributes as personal hygiene, emotional stability, enjoyment in school life, and the ability to cope with various circumstances [17].

All definitions state an outcome dimension of health literacy - an intermediate or distant goal or purpose of health literacy. Outcomes of health literacy include rather specific tasks linked to health-related decision-making, e.g. to manage one’s health environment [33] or to make informed or appropriate health choices [18]. The intermediate or long term outcomes of health literacy refer, rather generally, to the promotion of personal health [35] and health outcomes, e.g. the reduction of health risks and the improvement of ones quality of life [20] or living conditions [18]. Borzekowski [1] perceives children and young people as vulnerable and “marginalized” groups that can be empowered to be more engaged, more productive, and healthier. Paakkari and Paakkari [8] emphasise the societal dimension of health literacy, indicating that health literacy enables students “to work on and change the factors that constitute their own and others’ health chances”. Gordon et al. [19] take an even more general view, stating that health literacy is building individual and community capacity to understand the components of health. The categories “requirement” and “time perspective” are the ones that specifically relate to the target group of children and young people: The first includes preconditions for being (able to be) health literate, namely, a reasonable degree of autonomy [17] or supporting external conditions, as the ways health-related materials are presented in an age appropriate manner, are culturally relevant and socially supported [1]. Lastly, health literacy is viewed as being an evolving construct or ongoing process [19], which needs to be acquired and developed during the life course [10, 20, 34].

### Models of health literacy in children and young people

A total of 21 articles included models of health literacy (Table 4). These 16 articles conceptualized health literacy at a theoretical/abstract level. The other five operationalized health literacy dimensions for the development of measurement tools [18, 32] or as an effect or evaluation model for an intervention programme [22, 25, 26, 41]. Three models represented a clinical-medical perspective [21, 23, 33], but the majority of them (*n* = 18) took on a public health perspective. These studies developed health literacy from a multi-system perspective (i.e. the health system, education system, community system), covering several health-related domains, as health care, disease prevention and health promotion. Nine models were developed from a school health education perspective [8, 18, 22, 25–27, 29, 30, 32]. Three articles [25, 26, 32] included children younger than 12 years, while nine addressed young people or secondary school children aged 12 years or older. One article, Sanders et al. [5] covered four distinctive developmental phases. Eight studies did not exclusively focus on children and young people but considered health literacy over the life course.

**Table 4:** Models of children’s and young people’s health literacy (grouped according to target group and alphabetical order)

No	Author	Target group (TG) as expressed by papers	HL definition (Source: primary = by same author as model); secondary = different source)	Primary purpose of study	Context in which or for which developed/tested	Models described specificities of TG	TG participation in development	Empirically tested?
Children & Primary and Lower Secondary School Students								
1	Brown et al. [32] Exemplary for the US NHES [37]	Students, grade 5-8, Age 9-13	Primary (see Table 2)	Operationalization: Conceptual base for measurement tool	(School) health education	Yes	No	Yes
2	Paek et al. [28]	Students, grade 7, Age 12-13	Secondary (WHO, 1998)	Conceptualization; explorative & testing	Health Socialization	Yes	Yes	Yes
3	Schmidt et al. [25]	Children, grade 5, 9–13 years	Secondary (Nutbeam, 2008)	Operationalization: Effect model for intervention study	(School) health education	Yes	No	Yes
Young people & Secondary School Students								
4	Manganello [21]	Young people, n.s.	Secondary (IOM, 2004)	Conceptualization; Explorative	Health care; Disease prevention	Yes	No	No
5	Massey et al. [33]	Young people, aged 13-17	Primary (see Table 2)	Conceptualization inductive and explorative	Health care system	Yes	Yes	No
6	Paakkari & Paakkari [8]	Students, n.s.	Primary (see Table 2)	Conceptualization; theoretical exploration	(School) health education	Yes	No	No
7	Rask, Uusiautti, Määttä, [29]	Secondary school students, aged app. 18-20	Secondary, WHO (1998), as cited by Mancuso (2009)	Conceptualization; inductive	(School) health education	No	Yes	No
8	Steckelberg et al. [22]	Students, grade 11, Age 16-18	Secondary (IOM, 2004)	Operationalization: Effect model for intervention study	School curricula programme	Yes	No	Yes
9	Subramaniam et al. [26]	Young people, aged 10-15	Secondary (NAAL, 2013)	Operationalization: Deductive for intervention testing & evaluation	School health programme	Yes	No	Yes
10	Wharf Higgins, Begoray, & MacDonald [27]	Students, grade 10 (Canadian system)	Secondary (e.g. Kickbusch, 2007)	Conceptualization; inductive and testing	School health education	Yes	No	Yes
11	Wu et al. [18]	Students, grade 10 (Canadian system)	Primary (see Table 2)	Operationalization: Conceptual base for scale development	School health education	Yes	No	Yes
Different age groups or considering a life course perspective								
12	Lenartz et al. [38, 69] DE	General population, empirically tested for young people 17-21.	Secondary (Soellner et al., 2009)	Conceptualization; inductive and testing	Different health related domains	No	No	Yes
13	Mancuso [34]	Population across the life course, n.s.	Primary (see Table 2)	Conceptualization; explorative	Different health related domains	No	No	No
14	Martin & Chen [24]	Population across the life course	None stated	Conceptualization; explorative	Impact factors on child health	No	No	No
15	Nutbeam [35]	General population, focus upon health education	Primary (see Table 2)	Conceptualization; explorative	Public health; different health related domains	No	No	Yes
16	Sanders et al. [5, 31]	Children at different ages	Secondary (IOM, 2004)	Conceptualization; explorative	Different health related domains	Yes	No	No
17	Soellner et al. [36] DE	General population, including young people	Primary (see Table 2)	Conceptualization; inductive and testing	Different health related domains	No	No	Yes
18	Sørensen et al. [10]	Population across the life course, n.s.	Primary (see Table 2)	Conceptualization; explorative & testing	Health care; Disease prevention; Health Promotion	No	No	Yes
19	Wolf et al. [23]	Population across the life course, n.s.	Secondary (IOM, 2004)	Conceptualization; Explorative	Health care	No	No	No
20	Zarcadoolas, et al. [4]	Population across the life course, n.s.	Primary (see Table 2)	Conceptualization; inductive and explorative	Public Health	No	No	No
21	Zeyer & Odermatt [30] DE	Students, n.s	Secondary (HCC-Lab, 2005)	Conceptualization; explorative	(School) health education/biology	No	No	No

#### How are target group specificities considered?

Twelve of the identified articles elaborated on children and young people’s distinctiveness towards adults and how these specificities are relevant for understanding health literacy in these age groups. However, most of these considerations remained on a very broad level, strongly incorporating an “external”, adult view on the target group’s situation and the relevance of health literacy for them. In summary, children and young people:

- are expected to understand increasingly complex health information [5] and large amount of educational materials distributed to them by health providers, schools and intervention programmes [21];
- become increasingly responsible for their own health and for dealing with different kinds of health-related issues [22];
- are increasingly engaged in their health, their health service utilization [23] and usage of insurance benefits [33];
- develop skills today that influence their health (outcomes) and well-being over their life course [24] and reduce health expenditures [33];
- are citizens in their own right, within their current surroundings [8];
- are able to construct their own views on health matters [8, 35];
- are at a crucial stage of development characterized by many physical, emotional and cognitive changes [21].

Most prominently, articles considered children and young people’s situations and needs by exploring their social embeddedness, namely the interrelated pathways between the individual and their close and distal social contexts. Wharf Higgins et al. [27] stated that in order to be effective approaches to teaching health literacy “also need to reflect a thorough understanding of the structure of adolescents’ social worlds, and their developmental appropriateness”. While, Wharf Higgins et al. [27] reflected on health literacy from a socio-ecological understanding, Paek et al. [28] complemented the social ecological approach with health socialization perspectives, adopted from political and consumer socialization. As pathways of contextual influences are considered to be strong influencing factors of health literacy in the literature, an extensive description of the inductive content analysis is provided in the “antecedents and consequences” section. Moreover, the importance of an age- and development-specific understanding of health literacy for children and young people was especially pointed out in models that were developed within the context of school health education [18, 22, 25–29, 32]. Paakkari and Paakkari [8] stated that while health literacy learning conditions in school may include aspects of each of their five core health literacy components, students’ age-specific needs and characteristics need to be taken into account. The identified health education models conceptualized health literacy for a small and distinctive age group or specific school grade(s). Commonly, the complexity and comprehensiveness of their health literacy components increased by school grades. From a health promotion perspective, Sanders et al. [5, 31], similar to Borzekowsik [1], explored the development of health literacy competencies from a cognitive development perspective for different age groups. They distinguish between four successive developmental stages, providing examples of health literacy skills in four categories (prose/document and oral literacy, numeracy and system-navigation skills) that were adopted from the US National Health Education Standards (NHES) [37].

#### Dimensions of health literacy for children and young people in the 21 models

Health literacy in children and young people is described in the literature as comprising variable sets of key dimensions – clusters of related abilities, skills, commitments, and knowledge that enable a person to approach health information competently and effectively and to derive at health-promoting decisions and actions. This section provides an overview of the inductive content analysis which reveals the important aspects of health literacy in children and young people (Table 5). It also offers a meta-perspective of health literacy in children and young people that enables comparison between different aspects. As the retrieved dimensions are diverse and overlapping, classification was challenging. Due to the strong focus on individual attributes, the dimensions were selected to be clustered according to three core categories: (1) cognitive, (2) behavioural or operational and (3) affective and conative.

**Table 5:** Type and description of health literacy dimensions for children and young people

No	Author	Dimensions of HL (Skill, knowledge, …)	Nature/understanding of HL, as described in article
Children & Primary and Lower Secondary School Students			
1	Brown et al. 2007, [32] Exemplary for the US NHES [37]	(1) Critical thinking and problem solving, (2) responsibility and productivity, (3) self-directedness, and (4) effective communication Health literacy was operationalized in measurement as the ability - to comprehend concepts related to health promotion and disease prevention - to access valid health information - to demonstrate the ability to advocate for health by sharing information	Applies National Health Education Standards (NHES) in measurement instrument
2	Paek et al., 2011 [28]	Interests in health topics, Understanding of health subjects, Motivation to act on what they had learned about staying healthy	The model integrates perspectives from social ecological models and health socialization models, adopted from political and consumer socialization
3	Schmidt et al. 2010 [25]	Health literacy domains, which were operationalized in measurement:: Health knowledge, Communication, Attitude, Behaviour, Self-efficacy	Health literacy was assessed a construct consisting of the five dimensions mentioned; dimensions were based on author’s understanding of Nutbeam (2000);
Young people & Secondary School Students			
4	Manganello 2008 [21]	Functional literacy (basic ability to read and write) Communicative/interactive literacy (ability to participate in daily activities and communication Critical literacy (person’s potential to evaluate information) Media literacy (the ability to critically evaluate media messages)	Based on author’s understanding of Nutbeam (2000) & Kaiser Family Foundation (2006)
5	Massey et al. 2012 [33]	Navigating the system (e.g. to access service, understand (non-)emergency care, to make appointment & fill a prescription) Rights and responsibilities (self-care perceptions, asking questions, knowing rights regarding sensitive topics) Preventive care (well-care visit, screening behaviours, related attitudes/perceptions to visit doctor) Information seeking (Ability to evaluate information, passive and active information seeking, relevance of information provided by physician) Patient–provider relationship (perceived trust, comfort level, communication issues, continuity of provider care) Cross-sectional: knowledge, attitudes, practices within the health care setting	Focus on health care setting: Identification of dimensions was based on results from focus groups with young people & interviews with primary care physicians
6	Paakkari & Paakkari,2012 [8]	Health literacy as broad range of knowledge and competencies: Theoretical knowledge (explicit, factual, formal and declarative knowledge about health matters) Practical knowledge (procedural or skills-based knowledge; capability to do something) Critical thinking (ability to think clearly and rationally; e.g. understand health issues in deeper way, make sound choices, participate as active member of society) Self-awareness (ability to self-reflect on oneself, own decisions, and oneself as learner; Citizenship (ability to act in an ethically-responsible way and take social responsibility; consider health matters beyond one’s own perspective (the ones of others and of the collective) Cross-sectional skills: Basic reading, writing and speech skills social skills, communication skills, and information-gathering skills	Health literacy is defined as a learning outcomes of the school health education subject in Finland. It focusses on a health promoting and empowerment perspective with effects for the personal and societal health.
7	Rask, Uusiautti, Määttä, 2013 [29]	Basic/Functional literacy (basic ability to read and write) Communicative/interactive literacy (ability to participate in daily activities and communication Critical literacy (person’s potential to evaluate information) Extended with fourth dimension: Holistic health literacy, with four sub dimensions: - tolerance, - understanding culture as wide and multidimensional phenomena, - environmental consciousness, analysis of the state of the world from various points of view	Based on author’s understanding of Nutbeam’s typology (2000), complemented with own holistic health literacy models (adapted from Stewart et al. 2008), dimensions are elaborated from students’ perspectives & point of views, based on their answers from the Finnish Matriculation Examination
8	Steckelberg et al., 2009 a&b [22, 41]	Principles of critical health literacy related to evidence-based medicine (EBM): Understanding medical concepts (e.g. informed choice in diagnostic tests; appraising patient information) Skills of searching literature (e.g. searching the internet and databases) Basic statistics (e.g. critical appraisal of RCT’s) Design of health experiments and sampling (e.g. Fallacies and misinterpretations of data representation: observational articles versus randomized controlled trials (RCT) – What are the differences?; Understanding systematic reviews)	Based authors understanding of Nutbeam’s critical literacy dimension
9	Subramaniam et al. 2015 [26]	Health literacy skills inventory with main categories: Foundational elements: general abilities/characteristics (e.g. health-related knowledge, ability to listen, communicate, motivation, attitudes, intention & self-efficacy), access to information Health-related information: information need identification and question formulation, information search, information comprehension, information assessment, information management, information use (dependent on context/goal of health information seeking)	Integration of results from an own narrative literature review on definitions and models
10	Wharf Higgins et al., 2009 [27]	Skills to access, understand, and communicate health information, resources and services Cross-sectional skills: knowledge, attitude	Integration of results from a literature review on general health literacy dimensions, reflect a socio ecological understanding and health promotion perspective
11	Wu et al. 2010 [18]	Understand health information: - literacy types (prose, numeracy and document) & levels of difficulty. Evaluating health information: - result is a judgment or conclusion about the information that is presented; and this judgment or conclusion is based on applying one or more criteria:(1) accuracy, (2) impartiality, (3) relevance, (4) comprehensiveness, (5) internal consistency	
Different age groups or considering a life course perspective			
12	Lenartz et al. 2014; 2012 [38, 69] (Translated from German)	*Basic skills:* - health-related basic knowledge (Comprehension of basic terms describing the body or simple health-related coherences and functions) - health-related basic skills (health-related reading, writing and numeracy skills) *Advanced skills:* - perceptual-motivational level: health-related self-perception, acceptance of responsibility (Verantwortungsübernahme) - action-oriented level: (a) dealing with health information, (b) self-control, (c) self-regulation, (d) communication and cooperation. [translated]	Integration of the model by Soellner et al. (2009); models were tested in and evaluated by four different population samples
13	Mancuso, 2008 [34]	*Attributes of health literacy:* Capacity (skills and innate potential of individual, including gathering, analysing and evaluating health information for credibility and quality, working together, managing resources, seeking guidance and support, developing and expressing a sense of self, creating and pursuing a vision and goals, and keeping pace with change; oral language skills, social skills, reading, listening, analytical, decision-making and numerical abilities, ability to act on health information etc.) Comprehension (complex process based on effective interaction of logic, language, and experience; what the individual understands; identify and creatively address health issues) Communication (message or information exchange process, including speech, signals, writing or behaviour and involves input, decoding, encoding, output and feedback. Essential skills are reading with understanding, conveying ideas in writing, speaking so others can understand, listening actively, and observing critically)	Health literacy as competence: to have the capability to function effectively in health-care situations; explored from an education, library science, health care, public health and mental health context.
14	Martin & Chen, 2014 [24]	reading, writing, numeracy (ability to understand basic probability and numerical concepts), listening (aural language), and speaking (oral language) skills within a health context	
15	Nutbeam, 2000 [35]	Basic/Functional health literacy (basic skills in reading and writing to function effectively in everyday situations) Communicative/Interactive health literacy (more advanced cognitive and literacy skills which, together with social skills, can be used to actively participate in everyday situations, extract information and derive meaning from different forms of communication, and apply this to changing circumstance) Critical health literacy (more advanced cognitive skills which, together with social skills, can be applied to critically analyse information and use this to exert greater control over life events and situations) Foundational: Cognitive development, exposure to different information/messages (communication content and message), personal and social skills, self-efficacy	Focus on a health promoting and empowerment perspective.
16	Sanders 2009, a&b [5, 31]	Prose/document-literacy skills (Writing, text comprehension, filling out form) Oral literacy skills (from basic communication to negotiation) Numeracy skills (e.g. food portion size or to understanding screening results) Systems-navigation skills (describe media influence on behaviour to complete enrolment process) Foundational: collective of family health literacy skills is important (child, parent(s), other caregivers)	Integration of results from a own systematic literature review on child health and literacy; Adapted from NHES & Bright Future Guidelines; skills are individually composed; complexity increases with age (examples are provided for age 4, 10, 14 and 18)
17	Soellner et al., 2010 [36] (Translated from German)	Foundational skills (literacy/numeracy), Operational competence: (1) to navigate and act in the health system, (2) communication and cooperation, (3) information retrieval and processing, (4) self-awareness and self-regulation. Knowledge component: (1) system-specific und (2) health-related knowledge, Motivation: (1) willingness to take responsibility for own health. [translated]	Integration of results from an own narrative literature review on models and a stakeholder concept mapping
18	Sørensen et al.,2012 [10]	Access (ability to seek, find and obtain health information); Understand (ability to comprehend the health information that is accessed); Appraise (ability to interpret, filter, judge and evaluate the health information); Apply (ability to communicate and use the information to make a decision to maintain and improve health In three health domains: health care, disease prevention, health promotion Cross-sectional: knowledge, competence and motivation	Integration of results from an own systematic literature review on definitions and models
19	Wolf et al. 2009 [23]	Cognitive skill sets (processing speed, memory, reasoning and attention; literacy & numeracy, verbal ability and reading) Psychosocial skill set (self-efficacy, communication and prior experience)	Focuses on Health literacy as learning capacity in the health (care) setting.
20	Zarcadoolas et al., 2005 [4]	Fundamental literacy/numeracy (printed and spoken language, numerals, and basic mathematical symbols and terms) Science and technology literacy (e.g. knowledge of fundamental health and scientific concepts, comprehend technical complexity) Community/civic literacy (knowledge about sources of information and agendas, enables citizens to engage in dialogue and decision-making, includes media literacy skills and knowledge of civic and governmental processes) Cultural literacy (recognizing and using collective beliefs, customs, world-views, and social identity)	
21	Zeyer & Odermatt, 2009 [30] (Translated from German)	Situation-specific knowledge (to realize health-relevant situations, to match information) Conceptual knowledge (to realize health-relevant principles, facts, terms) Operational knowledge (knowledge on how to adequately act in an situation) Evaluation of health promotion (Does this action promote health and is feasible in daily life?) Evaluation of personal consequences Self-reflection (to break up every day routines and to consider and analyse one’s own action plan)	Health literacy is regarded as a set of competences.

##### Cognitive attributes

The mental abilities and actions that enable a person to think, learn and process information are attributed to this category.

- Knowledge

Knowledge is regarded as an essential component of health literacy in children and young people. Mancuso [34] states that a certain level of knowledge is required for comprehending content as well as for managing and analysing information and becoming empowered regarding one’s health and the related decisions. It is either described as (a) a separate core dimension [8, 30], as (b) an element of several dimensions [4], or (c) a foundational or cross-sectional component [10, 26, 27, 33]. Lenartz et al. [38] and Soellner et al. [36] describe health-related basic knowledge as the comprehension of basic terms describing the body or basic health-related coherences and functions. Others distinguish between (a) theoretical or conceptual knowledge (i.e. facts, terms, principles in health-related matters), (b) situation-specific knowledge (i.e. knowledge of specific health situations in health-related domains), and (c) practical or operational knowledge (e.g. the knowledge of what actions are adequate in a given situation) [8, 30]. Paakkari and Paakkari [8] describe conceptual knowledge as procedural knowledge or the skills needed “to behave in a health-promoting way” which is often experimental, situation-specific, and linked to daily practices. Massey [33] recognises that individuals must be knowledgeable and confident health care consumers. This includes the knowledge of one’s rights regarding sensitive topics, or knowledge of one’s responsibilities related to health care, e.g. health insurance benefits, how and where to find information.

- Basic or functional health-related skills

Most articles recognise that health literacy requires being able to read, write, fill out a form or comprehend a text [4, 21, 38]. Nutbeam [35] labels these skills as functional literacy which is needed in order to understand health-related materials (e.g. medicine labels, prescriptions, or directions for home health care) and to function effectively in everyday situations. Some authors point out the relevance of numeracy skills (e.g. the ability to understand basic mathematical symbols and terms, basic probability and numerical concepts) and active listening skills (aural language) [5, 24]. Wolf et al. [23] take on a cognitive development perspective, defining the mentioned health literacy skills as “higher order mental tasks”. The latter are determined by one’s (a) processing speed, (b) attention, (c) working memory, (d) long-term memory, and (e) reasoning (ibid. p.4). Consensus is lacking whether the described basic skills are considered as core dimensions of health literacy, or being integral in other dimensions, or rather preconditions for health literacy.

- Comprehension and understanding

The ability to comprehend, to grasps a meaning of and to understand health information or concepts related to health care, promotion and disease prevention was considered to be a core dimension of health literacy [10, 18, 28, 32]. Mancuso [34] refers to it as a complex process based on effective interaction of logic, language, and experience, allowing an individual to become a critical thinker and problem-solver who can identify and creatively address health issues. Subramaniam et al. [26] identified the following elements of comprehension: (a) an ability to read, comprehend and recall situated information; (b) an ability to perform basic mathematical functions (e.g. numeracy); (c) an ability to comprehend simple charts (e.g. visual literacy), and (d) an ability to filter information found and extract only relevant information.

- Appraisal and evaluation

The ability to interpret, filter, judge, and evaluate health information was another core dimension of health literacy [8, 10, 18, 21, 26, 29, 30, 32, 34, 35]. Moreover, appraising information refers to making sense of information gathered from diverse sources by identifying misconceptions, main and supporting ideas, conflicting information, point of view, and bias [26]. In the literature several relevant criteria but, as Wu et al. [18] stated, not necessarily mutually exclusive criteria for evaluating information, were identified: (a) accuracy, validity, and appropriateness (correct information or the message’s credibility); (b) impartiality (unbiased communication); (c) relevance (applicability to the problem); (d) comprehensiveness (broad coverage of the information); and (e) internal consistency (logical relationships exist between information and/or concepts). The credibility of the sources of a message or information, as well as the medium through which it is transported is also important [18, 21, 26]. Manganello [21] stresses that “media have been shown to influence physical and social development of youth, have been associated with health behaviour and are often cited as a source of health information for adolescents”. Zeyer and Odermatt [30] consider the evaluation of possible alternatives for action with regards to whether an action is health promoting and feasible in daily life. Hence health literacy entails evaluating the personal consequences of acting in a certain ways and the consideration concerning whether and how an intended action is feasible.

- Critical thinking

Critical literacy skills or critical thinking are argued to be core dimension of health literacy [8, 29, 35]. They refer to the ability to think clearly and rationally and approach knowledge from various angles, formulate arguments, and make sound decisions [8]. As children and young people receive health messages from numerous sources, “they may gain a fragmented picture of health issues unless they are able to critically create links between diverse pieces of information” [8]. As a result, “critical thinking enables students to deal with large amounts of knowledge and to have power over that knowledge” [8].

##### Behavioural or operational attributes

All dimensions referring to actions that take place outside of the individual’s mind were assigned to be behavioural attributes.

- Seeking and accessing information

Information seeking is described as another core dimension of health literacy. Subramaniam et al. [26] view it as a fluid and iterative process, including two main elements, namely information access and search. Accessing information is the ability to seek, find and obtain health information [10]. According to Subramaniam et al. [26] it includes being able to adapt to new technologies, being aware of primary health resources to begin search, having to access valid information, products, and services, being exposed to computers in everyday life and being aware of search engines and their capabilities. Massey [33] distinguishes between materials received from health providers (“passive information”) and information accessed over the Internet or by other means outside of the clinical setting (“active information”). Searching information entails developing appropriate search strategies, using relevant and correctly spelled search terms, applying an adequate search strategy drawing on reputed credibility and an understanding of how search engines work (e.g. hits, order of search results, snippets, inclusion/placement of ads, etc.). Moreover, Subramaniam et al. [26] included other elements such as being able to limit reliance on surface characteristics, among others the design of a website, the language used, etc. (e.g. surface credibility), to reduce search result selection based solely on word familiarity and to use translation features on the search engine or Web page if needed. As such critical media literacy and critical digital literacy have become important dimensions of health literacy in the information society. Moreover, Paek et al. [28] distinguish traditional media, such as TV, radio and newspapers, from digital media, e.g. the internet.

- Communication and interaction

Communication, according to Mancuso [34], refers to how thoughts, messages or information are exchanged and includes speech, signals, writing or behaviour. It further involves input, decoding, encoding, output and feedback. Being able to effectively communicate about one’s own health or health information and, if necessary, to cooperate with other people, including friends and health care providers was considered an important aspect of health literacy [23, 25, 27, 32, 36, 38]. According to Nutbeam [35], more advanced cognitive, literacy and social skills are needed in order to “communicate in ways that invite interaction, participation and critical analysis”, to extract information and derive meaning from different forms of communication, and to apply this to changing circumstances [35]. Basic communication about health (issues) in health care settings requires providing an overview of personal medical history or participating in informed consent discussions about medical treatment options [33]. Essential communication skills involve reading with understanding, conveying ideas in writing and speaking so others can understand, listening actively, and observing critically [34]. Moreover, young people and children need listening (aural language) and oral literacy or verbal/expressive skills in order to effectively communicate [5, 26]. While Paakkari and Paakkari [8] recognise that health literacy involves being able to “clearly communicate one’s ideas and thoughts to others”, they regard general communication and social skills as foundational for health literacy and not as a distinctive dimension of health literacy.

- Application of information

This core aspect of health literacy refers to the ability to communicate and use health information for health-related decision-making with the rational that one wants to maintain and improve one’s health and that of the people in one’s surrounding. The use of health information strongly depends upon the context and the goal of the health information seeking process [26]. It entails being able to synthesize information from multiple sources, draw conclusions, answer questions originally formulated to present information need, or even sharing, collaborating, communicating, creating information and adapting them as needed for intended audience (e.g. self, peers, family). On an outcome or impact level, applying health information refers to addressing or solving health problems, and make health-related decisions. This includes using health information for practicing health-enhancing behaviours or mitigating or avoiding health risks. Massey et al. [33] focus on young people’s health prevention behaviours, such as participating in annual check-ups or screening interventions as well as their attitudes and perceptions about visiting a doctor. On a population level, applying health information entails advocating for personal, family, and/or community health [8, 26]. From a critical scientific perspective, it includes being able to interpret data of scientific articles to articulate potential limitations of published research findings and the cumulative impact of scientific knowledge (i.e. incremental process of discovery), as well as being able to recognise inaccurate information and to practice appropriate ethical standards for information (e.g. copyright, security, privacy) [22, 26].

- Other context-specific skills for the application of information and accessing services

The ability to navigate through the health care system was defined as a core dimension of health literacy [36]. It entails knowing how to access health services and being able to make an appointment or filling out a prescription [33]. Sanders et al. [5] provide age-adjusted examples for navigation skills which range from knowing proper usage of emergency numbers (e.g. 911) for school aged children to accessing confidential health and counselling services (young people) or completing enrolment processes for a health insurance and obtaining appropriate health services (young adults, 18-20 years).

- Citizenship

Citizenship, the ability to act in an ethically-responsible way and take social responsibility, defines a core dimension of health literacy in the work of Paakkari and Paakkari [8]. It involves considering health matters beyond one’s own perspective, namely through the lens of others and of the collective, as well as moving from individual behaviour changes towards wider changes (i.e. organisational changes). Similarly, Zarcadoolas et al. [4] consider civic literacy a core dimension of health literacy. It describes the “knowledge about sources of information, and about agendas and how to interpret them, that enables citizens to engage in dialogue and decision-making”. Rask et al. [29] take on a societal perspective in their holistic health literacy dimension by identifying particular skills: People who possess holistic health literacy are (a) tolerant to various groups of people, (b) antiracist, (c) widely aware of the influence of cultural differences on health, (d) aware of the importance of art and civilization for health, (e) concerned about the environmental threat. Moreover, they (f) understand the significance of social capital for physical, mental, and social health, (g) appreciate and protect environment, (h) criticize the negative aspects of western life because they pose a threat to health, and (i) want to promote health globally.

##### Affective and conative attributes

This category includes dimensions of health literacy that evolve around the experience of feeling or emotions (affective attribute) or describe personality traits and mental stages that influence how individuals strive towards action and direct their efforts (conative attributes).

- Self-awareness and self-reflection

Self-awareness involves the ability to reflect on oneself. It refers to being conscious about one’s thoughts, feelings, attitudes, values, motives and experiences as well as one’s health-related decisions [8]. “Self-awareness requires being able to link together and describe health topics from one’s own personal perspective, and to examine reasons for one’s ways of behaving and thinking in a particular way”. An adequate perception of one’s needs, wants and sensations is seen as key factor for regulating one’s own behaviour [36, 38] and for breaking through daily-routines and considering and analysing a strategy for action [30]. Paakkari and Paakkari [8] also stress the ability to reflect oneself as a learner, namely the ability to evaluate their learning strategies, define learning goals, and monitor their progress.

- Self-control and self-regulation

According to Lenartz et al. [38], self-regulation enables individuals to formulate health-related goals in line with as many personal needs, feelings, values, and interests as possible. Self-control refers to an inner focus to reach a certain goal, while possibly struggling with competing personal needs, feelings, wishes and interests. A certain level of self-control and self-regulation is therefore needed to resist the internal and external (social) pressure (e.g. to continue or start smoking again) and to deal with e.g. unpleasant feelings and emerging doubts [36, 38].

- Self-efficacy

Self-efficacy – a person’s own belief in their own ability to complete certain health-related tasks and reach defined goals was considered a foundational dimensions of health literacy in children and young people [23, 25, 26, 35].

- Interest and motivation

Young people’s interest in health topics and their motivation to act upon what they have learned in staying healthy were described by Paek et al. [28] as core dimensions of health literacy. Similarly, Sørensen et al. [10] regard motivation as an essential cross-sectional component, and Soellner et al. [36] emphasize the willingness to take responsibility for one’s own health.

#### Antecedents and consequences of health literacy in children and young people

Table 6 displays the factors that the literature review identified as influencing children’s or young people’s health literacy (antecedents) or as being influenced by health literacy (consequences).

**Table 6:** Antecedents and consequences of health literacy in children and young people (models that did not state any are not shown)

No	Author	Factors that influence the development and maintenance of HL	Factors that are influenced by Health Literacy or impact on the relationship between HL & health outcomes
Children & Primary and Lower Secondary School Students			
2	Paek et al. 2011 [28]	Demographics & Environments (Gender, Ethnicity, Residence (rural/urban), health status, environmental risk factors, access to health information. Socialization Process: - socialization agents a) interpersonal channel (parents, peer, school) vs. b) Media (push media: TV, Radio, Newspaper; pull media: Internet), - health information behaviour, frequent consumption of health information, source of health information	(Health) behavioural outcomes
3	Schmidt et al. 2010 [25]	n.m.	health behaviour (as intermediate health outcome)
Young people & Secondary School Students			
4	Manganello, 2008 [21]	- individual traits/characteristics: such as age, ethnicity, gender, cultural background, cognitive and physical abilities and social skills; - media use - peer and parental influences: home setting, parental (health) literacy; - systems: media, education and health care	Health behaviour: e.g. to be informed and skilled health care consumers, Health service use: e.g. to effectively navigate the health care system & use health insurance benefits Health costs
5	Massey et al. 2012 [33]	n.m.	- health environment requires individuals to be informed and skilled HC consumers, - overcome environmental & interpersonal barriers when interacting with the HC system
6	Paakkari & Paakkari, 2012 [8]	Learning process and learning environment/conditions: teaching methods for health literacy need to be age-adjusted, pupil-focused, reflective, through discussion and negotiation	Empowerment, be able to understand oneself, others and the world, make sound health decisions, contribute to changing the factors that impact one’s own health and the health of other
7	Rask, Uusiautti, Määttä, 2013 [29]	poverty, gender, cultural differences, level of education, social economic status	knowledge & skills in maintaining their own health, ability to discuss health-related social issues
10	Wharf Higgins et al., 2009 [27]	Mico context:- Internal influences: age, gender, beliefs, values, experiences, SES,- General literacy (ability to read/write, listen/speak, view/represent; - other abilities, e.g. technological abilities with information mediaMeso Context: - School, family, and peer factors affecting health (e.g. family SES, peer norms and behaviour, safe/healthy schools etc.)- Health curricula: teaching, assignments, activities, testing, resources, etc.Macro context: - External influences: societal, community and neighbourhood factors affecting health (e.g. community-level SES, culture, media, government policies, etc.).	To establish and maintain an individual’s health-related goals (e.g. do not drink before driving, to exercise)
Different age groups or considering a life course perspective			
12	Lenartz et al. 2014; 2012 [38, 69]	n.m.	Health behaviour and health
13	Mancuso, 2008 [34]	*Competences:* Operational (ability to utilize tools, procedures, and techniques for handling language proficiently), - Interactive (collaboration with others for individual improvement & enhancement through self-management.), - Autonomous (personal empowerment & self-awareness), - Informational (ability to determine authority and the currency of health information), - Contextual (mastery of the (health care) environment), - Cultural (ability to interpret the meaning system of social practices)	Healthcare costs, knowledge of diseases and treatments, self-management skills, ability to care for chronic conditions, compliance, medical or medication treatment errors - Access to and use of healthcare services - Use of expensive services such as emergency care and inpatient admissions - Prevention, screening, and health-promoting behaviours - Health status, defined as physical illness or perceptions of illness, disease or impairment
14	Martin & Chen, 2014 [24]	- parental health literacy & parental SES, health and health behaviour influence children health, school readiness, and academic outcomes; informal home setting, with downstream effects in formal academic setting; System influences and potential intervention point: - health and education setting/system, culture and society,	child HL influences HL as parents, parental SES, health, and health behaviours
15	Nutbeam, 2000 [35]	Health promotion actions: - Education (e.g. patient & school education, broadcast media and print media communication), - Social mobilization (e.g. community development, group facilitation, targeted mass communication), - Advocacy (e.g. lobbying, political organization and activism, overcoming bureaucratic inertia)	Individual benefits - Greater autonomy and personal empowerment - Improved knowledge of risks and health services - Compliance with prescribed actions. - Improved capacity to act independently on knowledge - Improved motivation and self-confidence - Improved individual resilience to adversity Community/social benefits - Increased participation in population health programmes - Improved capacity to influence social norms and interact with social groups. - Improved capacity to act on social and economic determinants of health - Improved community empowerment
16	Sanders et al. 2009 a [31]	Family factors: income, education, language, culture, social support; Social factors: geography, educational resources, public health support, environmental health Different systems: - Educational system (Preschool, K-12 curricula, adult education/job training), - Community systems (after-school programmes, culture/language, public health programmes), - Patient care environment (Provider skills, information tools), - Health systems (delivery system, information system)	Family health behaviours, Child health outcomes
18	Sørensen et al.,2012 [10]	Distal factors: - Social and environmental determinants (e.g. demographic situation, culture, language, political forces, societal systems); Proximal factors: - personal determinants (age, gender, ethnicity, socioeconomic status, education, occupation, employment, income, literacy), - situational determinants (e.g. social support, family and peer influences, media use and physical environment).	health service use, health costs, health outcomes, health behaviour, participation, empowerment, equity, sustainability
19	Wolf et al. 2009 [23]	n.m.	Health knowledge, health behaviour, health outcomes
20	Zarcadoolas et al., 2005/ 2003 [4]	Health status; Demographic, socio-political, psychosocial and cultural factors	Ability to apply information, to participate in public and private dialogues about health, medicine, scientific knowledge and cultural beliefs

##### Antecedents

Twelve of the identified models included antecedents and distinguished between individual characteristics, demographic, situational or contextual factors as well as broader system or social factors.

Internal characteristics refer to an individual’s beliefs, values, experiences, cognitive and physical abilities, general literacy skills or other abilities, e.g. technological abilities. Paakkari and Paakkari [8] argue, in line with Manganello [21], that general skills such as social or communication skills, as well as self-efficacy are antecedents for health literacy and not per se separate core dimension of health literacy. Rather, they are important for different core dimensions and are not attributable to one. However, other authors [35] regard these as being core dimension of health literacy (see Table 5).

Models that focus specifically on children and young people emphasize the family’s demographic factors and parental influences. The younger the child is the more likely he/she is to rely on their parents for economic and social support and, therefore, their own socioeconomic status (SES) or occupation are not applicable to variables [24, 31]. Family demographic factors include parental health literacy levels, socio-economic status, as well as their own health status and health behaviour. Martin and Chen [24] argue that these family factors strongly influence children’s health literacy, health status, and other educational variables such as school readiness and a child’s academic outcomes.

Furthermore, families, peers, and schools are all regarded as major socialization agents in children’s and young people’s lives that influence the opportunities they have for being or becoming health literate. Family and peers can encourage or discourage health literacy actions as well as health promoting lifestyles through their norms, actions, and social support [27]. Parents can be role models of how to access and interpret health information and teach children to critically evaluate the credibility and validity of information sources and media channels. In this context, the quality and the type of the relationship play a major role, as children or young people are likely to consult peers and adults they trust, which is crucial as trust also plays a role in accessing media and online health resources.

The social and system levels refer to education, health, and community systems as well as political and cultural forces. These include the general learning conditions and environment, e.g. students’ safety on school grounds, teachers that are equipped with the appropriate skills and teaching practices that could promote critical thinking and reflexion through negotiation and discussion [8, 27]. Next, the community where a child or young person lives may have an impact on his/her health literacy: Martin and Chen [24] and Wharf Higgins et al. [27] draw attention to the influence of the community- socioeconomic level on the health literacy in that community. Political and cultural factors refer to differences in cultural practices, political decision-making, e.g. governmental policies that decide whether to include health literacy in the school health curriculum. Synthesizing it, health literacy is argued to be promoted through health promotion actions in the general population which include an education for health, efforts to mobilize people’s collective energy, resources, skills, towards the improvement of health and advocacy for health, e.g. in form of lobbying activities and political activism [35].

##### Consequences

Fifteen articles mention that health literacy in children and young people leads to benefits on the individual, community or societal-level (applied from Nutbeam [35]). On individual level, health literacy enables young people to be skilled health care consumers and to overcome environmental and interpersonal barriers when interacting with the health care system [21, 33, 35]. Moreover, it is argued that health literacy can empower young people to understand themselves, others and the world, to make sound health decisions, and to discuss health-related social issues [8, 29]. Health literate young people are also believed to possess an enhanced ability to establish and maintain their self-defined health-related goals such as to engage in physical activities or not to drive after drinking [27]. In addition, the benefits of health literacy are argued to extend to the full range of life’s activities – at home, at work, in society and culture and at wider health economic levels [4, 10]. Martin and Chen [24] and Sanders et al. [31] take on a life course perspective, viewing health literacy as set of competencies that are passed from a parent to the child and do not only affect the child’s health behaviour and outcomes but also the ones of the family.

In terms of societal and communicational benefits, health literacy is argued to increase the participation in population health programmes, to improve community empowerment and the general capacity to influence one’s own health and the health of others, as well as broader social norms [8, 35].

## Discussion

The objectives of this study was (a) to scope current understandings of health literacy in childhood and youth and (b) to understand to what extent available models capture the unique needs and characteristics of children and young people. The 12 definitions and 21 models identified enabled a sound depiction of health literacy for children and young people. As a strong commonality of the complex and heterogeneous definitions and models, health literacy is depicted as a multidimensional, complex construct. Moreover, by describing the construct along multiple integrated categories, a synthesis of the health literacy dimensions retrieved from the literature was possible. However, it may be the case that these categories overlap as the same phenomena can be described in various ways and many models regarded health literacy through different lenses, resulting in differential focuses. These observations are in line with Paakkari and Paakkari [8] who pointed out that “there are differences regarding what is regarded as a component of health literacy and what may follow on from or be associated with health literacy”.

Regarding the first part of the research question, the focus of health literacy exceeds the health care setting in most definitions and models. It was recognised that health literacy in children and young people is relevant in many occasions and contexts of daily life that have a potential impact on the well-being and the promotion of one’s health. Similar to health literacy in adults [10], health literacy involves actions or agency which vary according to the health literacy perspective that is applied – e.g. from a clinical or health care setting paradigm, to a more comprehensive health system or public health or health promotion paradigm [42, 43]. While the first perspective aims to impact on the health outcomes of the individual through healthier decision-making, the latter includes actions for advocating for one’s own health and that of society through citizenship [8] and addressing broader social determinants of health [29]. These definitions and models are referred by De Leeuw [42] as “third generation” health literacy research which recognise that health literacy enables personal empowerment and is interrelated with broader determinants of health. As a result, health literacy is context and content-specific and as such varies according to the complexity of the task at hand and the contextual factors present [35, 43]. Hence, an individual is always interwoven with and subjected to the social and cultural context it is embedded. While these “two sides of a coin” – the individual’s attributes and the many contextual factors – were considered in most definitions and models identified, the review revealed a strong emphasis on the individual attributes which were elaborated in detail. The contextual factors were acknowledged but often remained underscored in the literature. In the following paragraphs, we offer our reflection and perspective on the observed discrepancy.

The individual attributes include the knowledge and skills that a person should have in order to meet certain situation-demands, e.g. in the health (care) system, or general health-related demands that society poses upon the individual. These demands mostly are diverse and overlapping within the definitions and models. Mostly, they refer to performing actions related to the gathering, understanding, appraisal and use of health information or services, or as Fok and Wong [17] point out, general physical and psycho-social activities. However, this individual-based, action-focused perspective “appears to limit the problem of health literacy to the capacity and competence of the individual” [44]. Moreover, the behavioural components of health literacy (e.g. to apply health information) are often not distinguishable from the outcome categories of health literacy, namely the health choices and behaviours that are health literacy is expected to influence (listed in Table 6). Given the strong individual and skill-based focus of health literacy definitions and models that require individuals to take charge of and become actively involved in seeking, understanding, accessing information and make health-related decisions, really reflects children’s and young people’s everyday realities. In other words, do they overestimate the opportunities (Möglichkeitsraum) and scope for action (Handlungsspielraum) of children and young people within health literacy and decision-making processes? According to Schulz and Nakamoto [45], health literacy and personal empowerment do not automatically derive from one another, as one can have the capacities and skills necessary to promote one’s health but may lack the empowerment to do so. Moreover, the preferred “societal” outcome of most models is “healthier behaviour” – namely such behaviour that is considered “healthy” by health professionals, experts or society. Especially models targeting the health care system still appear to strongly favour an adherence perspective, viewing individuals primarily as receiving health information and complying with the professional (health or care) instructions provided. Such strongly “subject-focused” health literacy perspectives entail – as known from health promotion discussions – the risk of primarily holding the individual responsible and accountable for their own health. This reflects a culture of individualisation in late modernity and “the risk society” [46, 47]. This victim blaming approach [48] ignores the universal recognition that social determinants of health – the economic and social conditions that affect individuals and communities –strongly influence a person’s individual ability to be health literate [11]. By ignoring the multifaceted and complex nature of human decision-making and behavioural change [49, 50] and by clashing with health promotion goals and practice, individual-level health literacy perspectives “may do little to achieve the ultimate goal of promoting equitable health status” [51]. As a result, exercising health literacy is only possible if opportunities for engaging in health literacy actions as well as for participating in everyday decision-making are present. Hence, the extent to which families, communities and societies allow children and young people to take an active role and participate in health literacy practices remains a question for future research. A possibility for exploring this could be by drawing upon a resource-focused health perspective, for instance the salutogenic paradigm by Antonovsky [52]. Saboga-Nunes [53] stressed the connectedness between health literacy and salutogenesis by arguing that childhood and youth could be considered most permeable life stages where salutogenic resources are built up by transforming health information into action and the other way around. In line with Antonovsky [52], health information could be understood as stimuli from one’s internal and external environment that are met with a dynamic feeling of confidence. This feeling would be retrieved from the ability to comprehend such stimuli, to consider them to be relevant for one’s health, and to access the resources needed for successfully addressing the stimuli and the demands posed by it.

In terms of the interrelatedness of social, cultural, and environmental contextual factors, especially the role of the intermediate environment of children and young people is emphasized: The target group is especially dependent upon their parents or caregivers for the access to material, financial, and social resources (e.g. health care). However, this dependence decreases as they develop and become more mature. While most articles also identify a strong impact of adults’ health literacy on their children, little is known about the nature of this influence and the impact of social agents in the target group’s environment. Sanders et al. [31] refer to it as “collective health literacy”, which can be regarded as a form of social and cultural capital according to Bourdieu [54]. Moreover, several articles highlight the role of available and accessible social support structures and peer assistance for the health literacy of children and young people: they benefit from the health literacy related knowledge and skills which they can access through their social informal or formal support structures. This kind of assistance can help children to accomplish health-literate-related tasks or actions that they otherwise would not be able to succeed in on their own [1, 55]. Vygotsky [56] termed this external assistance “scaffolding”. Overall, these social-cultural and economic contextual factors are primarily argued to act as antecedents or mediators for health literacy and tend to be neglected at the core of health literacy itself. We argue that the strong emphasis on health literacy as a set of skills tends to neglect and disregard the situation in which health literacy takes place, as well as the social practices relating to health literacy. In conclusion, there is a gap between the recognition of the role of contextual and cultural factors for health literacy and their implementation within strongly individualistic, skill-based conceptualisations, as well as operationalisations that focus on few distinctive health literacy dimensions [25]. Therefore, further research is needed that shifts from a functional, skill-based health literacy perspective to alternative approaches of understanding health literacy, e.g. by observing health literacy within the context that it takes place in and through the social practices in which it is performed. Such a comprehensive health literacy construct will be challenging to implement and operationalize. One option for addressing this challenge could be a modular design, which is then adjusted as necessary to specific target groups, contents and contexts.

The second part of the research question was to clarify to what extent available models capture the unique needs and characteristics of children and young people. Here, special attention was contributed to the target groups' recognition and characteristics in the analysis, which revealed the following discussion points:

While many definitions and models were identified for young people, including secondary school students, similar findings are lacking for children under the age of ten or within a primary school context. In addition, the same is true for transitional stages, e.g. from primary to secondary school level or from youth to adulthood. These findings are in line with conclusions drawn by Hagell, Rigby and Perrow [57]. Especially with regards to young children, the focus is strongly on maternal or caregivers’ health literacy competencies, enabling them to secure the child’s care needs. Children, including primary school level or younger have not yet been at the focus of health literacy conceptual and intervention research efforts. Given that research has linked health literacy to health outcomes, and to health (care) costs for the adult population, research should follow up on past efforts [58] in order to explore the relevance for young people as well as children.

Life phase specificities are only considered in 12 models, which incorporate a strong focus on children’s age-specific cognitive development. These dominantly consider health literacy to take place in several consecutive age or developmental stages, as Piaget suggested in his theory of cognitive development [59]. Although life phase specificities are argued to manifest in the target group’s social embeddedness, the articles attribute little attention to sociological approaches to childhood [54, 60] as well as to children and young people’s social role and position, as argued by the New Sociology of Childhood [61]. The latter perspective of childhood stresses that children should not be regarded as ‘becomings’ (incomplete) but as individual “beings” and members of their own social groups. This draws attention to the social role that is contributed to children and young people by their caregivers, communities and society. Generally, the younger children are the more dependent they are on their parents in respect to economic resources and social support as well as their parents’ health literacy. However, little is known about how parental and child health literacy are interwoven and interact in the child’s developmental processes. Brady, Lowe, and Lauritzen [62] for instance argue that even from a very young age onward, children are already active agents of their own social worlds that take on an active role in their health. Viewing children and young people as active social agents draws attention to considering children’s perspective of health and how they deal with it while being subjected to different social contexts and cultures. Children continuously develop and change through socialization processes and interaction with their environment, including their parents, other adults or their peers [61]. How we view children and young people, therefore, largely depends on our – adult – perception of childhood and youth and the social role we attribute to children and young people in everyday interactions, e.g. between teachers and students or between doctors and child patients.

The essential role of media and digital communication channels for the target group [63] was a theme that was found to remain underscored in available health literacy dimensions for children and young people, apart from few exceptions [21, 27, 28]. Media plays an increasing role in children’s personality, cognitive and emotional development. It transports moral and cultural values and facilitates their social and political socialization processes [64]. In an attempt to bridge the conceptual gap between approaches to health and media literacy, a media health literacy model for adolescents was developed and successfully tested for the target group by Levin-Zamir et al. [65]. Moreover, critical media health literacy for young people was defined by Wharf Higgins and Begoray [66] as consisting of a skill set of reflection, discrimination and interpretation abilities, as well as empowerment and engaged citizenship. Given the important role of media in the target group, we propose to recognise the interrelatedness of (critical) media, digital and health literacies more profoundly in future models, interventions, and educational curricula.

Most of the identified dimensions of health literacy in childhood and youth were fairly similar to the ones identified for adults (cf. the review results by Sørensen, et al. [10]). This poor incorporation of life phase specificities might result from the fact that their voices and perspectives largely remain unheard: Their active participation in the conceptual development process was only realized in three articles. Overlaps to adult health literacy dimensions were observed most strongly in models that focused on a life course perspective of health literacy (and hence implicitly target children and young people as well). Those six models were analysed to be adult-focused as they incorporate neither target group specifics nor age- or development-flexible components. Therefore, their applicability and validity for the target group was found to be questionable. This is especially problematic as they have served [67, 68] or may in the future serve as conceptual foundations for health literacy programs or interventions for children and young people. Applying general health literacy models to the target group that were not especially developed to meet the needs and demands of children and young people may actually hinder effective health literacy promotion and development in that target group. Such practices have been observed in some summarizing articles on children’s and young people’s health literacy as well [39, 57]. The described scarcity of health literacy understandings that incorporate specific target group characteristics and perspectives reveals a current research gap.

Therefore, it is argued in line with Rubene et al. [55] that children’s and young people’s health literacy, due to their distinctive needs and life situations, should be “conceptualized as an issue in its own right and not as a derivation of adult health literacy”. Hence, future conceptual and empirical research efforts need to recognise children’s and young people’s special character and encourage the target group to actively participate, providing them with the opportunity to contribute with their own understandings and perspectives of health literacy and to the promotion of healthy behaviour.

### Limitations

For pragmatic reasons, this review focused on exploring definitions and models of general health literacy of young people, excluding domain- (e.g. *media*), target-group or disease-specific health literacies (e.g. mental or diabetes health literacy). However, concentrating on generic health literacy enables a broader recognition of the overall field of health literacy, hopefully preventing us from ‘not seeing the wood for the trees’ due to specific interest areas [16]. Macket et al. [16] point out that a model valid for one context is less helpful for enabling knowledge construction and learning in other contexts through cross-contextual comparison and transfer. While this is an acknowledged problematic, we strongly stress the need to view health literacy as being socially constructed, varying according to the context one is in and the tasks at hand and hence recognising the unique characteristics of the target group.

Extending the review to articles that incorporate a life course perspective on health literacy may have let to bias the analysis towards non-target group-specific definitions and models. However, these were included based on the argumentation that if they claim to provide a life course perspectives on health literacy, they implicitly includes children and young people as well. Therefore, they are of relevance for the comprehensive scoping of current health literacy understandings for the target group. While the review was conducted using sound and systematic methods, following the PRISMA guidelines to the extent possible for qualitative reviews [14], in order to ensure its validity and accurateness, several limitations certainly are present and need to be considered. Efforts were made to enhance the sensitivity of the search strategy, using a comprehensive list of search terms and applying relevant operators. The databases that were used covered multiple disciplines indexing bibliographic records of a variety of journals and publication types. Nevertheless, we might have missed relevant literature, among others, due to limitations in availability and of individual databases’ search algorithms. Focussing only on English and German language articles led to distortion in favour of native English and German speaking research contexts. To ensure that the focus remains on the key research question, the assessment and evaluation of the selected articles was performed according to a systematic data extraction method, applying a coding protocol. While the core research team was independently involved in the selection and the assessment of the articles to minimize subjectivity and interpretation, the risk of selection, coding or opinion bias still remains. Due to the differing focus of analysed definitions and models, an explicit evaluation of the content was often difficult. Hence, the final assessment depended on the researchers’ interpretation of the written content. Furthermore, no assessment of the articles’ methodological quality took place, as many were theory-building or conceptual, explorative publications that often did not follow an outlined methodological approach. Therefore, not all quality standards as outlined in the PRISMA guidelines were applicable and viable for our research design.

## Conclusion

Addressing health literacy in children and young people should be based upon an empirical sound and measurable definitions as well as on conceptual frameworks that are valid, hands-on, and meet the specificities of the target group. This systematic review of the literature identified a broad theoretical base for health literacy in children and young people, while also pointing to conceptual shortfalls, especially related to a coinciding set of knowledge and skills adopted for the target group and how these are developed during the life course. Moreover, further operationalisation and implementation of these dimensions are necessary to test whether the described commonalities of the definitions and models are sound and measurable to describe the construct of health literacy of children and young people. Furthermore, we believe that health literacy could empower children and young people – who are especially vulnerable and to some extent marginalized social groups – to become more engaged with their health and more informed and reflective upon their future health choices. For this, it is crucial to not focus on an individualistic perspective only. Rather, it is of importance to recognize the interrelatedness and contextualisation of health literacy where people are empowered to interact with health, social and educational systems to the benefit for themselves as well as for the society as a whole. In turn organisations and systems are providing health literacy friendly services that can facilitate health for all. In such a two-sided approach, we must pursue to (i) strengthen children’s and young people’s and their care takers’ personal knowledge, motivation and competences to take well-informed health decisions; and (ii) decrease the complexity of society as a whole, and of the health care system in particular to better guide, facilitate and empower citizens, including children and young people to sustainably manage their health. Future efforts must target the redesigning of systems to be inclusive and friendly towards children and young people, the adjustment of curricula and training of health professionals, teachers and other relevant stakeholders in order to better meet the challenge of the health literacy deficit, and the recognition of children and young people as active partners in their health decision-making.

Moreover, given the relevance of social structures and support on the way health literacy skills are acquired, applied and hence practiced in very varying life situations, children’s and young people’s distinctiveness from adults, however, should become a crucial consideration when understanding health literacy. Moreover, we stress that health literacy should not become a liability for children and young people with responsibilities exceeding their influence. Hence, several critical reflections and considerations that challenge current understandings of health literacy were pointed out that could be beneficial when taken into account in future research and interventions. Therefore, future efforts should encompass these gaps and challenges identified, addressing them from a multidisciplinary perspective, viewing the target group as active social agents, who are deeply embedded in their close and distant surrounding (e.g. family, friends, and social institutions). As such, the greatest challenges for conceptualizing health literacy might ensure its generalizability and validity across context, while recognising its context- and content-dependency.
