# Erratum to: Health literacy in childhood and youth: a systematic review of definitions and models

**DOI:** 10.1186/s12889-017-4365-x

**Published:** 2017-05-09

**Authors:** Janine Bröder, Orkan Okan, Ullrich Bauer, Dirk Bruland, Sandra Schlupp, Torsten M. Bollweg, Luis Saboga-Nunes, Emma Bond, Kristine Sørensen, Eva-Maria Bitzer, Susanne Jordan, Olga Domanska, Christiane Firnges, Graça S. Carvalho, Uwe H. Bittlingmayer, Diane Levin-Zamir, Jürgen Pelikan, Diana Sahrai, Albert Lenz, Patricia Wahl, Malcolm Thomas, Fabian Kessl, Paulo Pinheiro

**Affiliations:** 1 Centre for Prevention and Intervention in Childhood and Adolescence CPI, Bielefeld University, Bielefeld, Germany; 2 National School of Public Health, Universidade NOVA de Lisboa, Lisbon, Portugal; 3 University of Suffolk, Ipswich, UK; 4 Global Health Literacy Academy, Urmond, The Netherlands; 5 University of Education, Freiburg i.Br, Germany; 6 Robert Koch Institute, Berlin, Germany; 7 CIEC, Institute of Education, University of Minho, Braga, Portugal; 8 School of Public Health, University of Haifa, Haifa, Israel; 9 Austrian Public Health Institute, Gesundheit Österreich GmbH, Wien, Austria; 10 School of Education, Basel, Switzerland; 11 Katholische Hochschule Nordrhein-Westfalen, Paderborn, Germany; 12 School of Education and Lifelong Learning, Aberystwyth University, Aberystwyth, UK; 13 University Duisburg-Essen, Essen, Germany

## Erratum

Following publication of this article [1], it has come to our attention that one of the authors has had their name misspelt. “Ullich Bauer” should actually be read as “Ullrich Bauer”. The original article has been updated to reflect this.
